# What Is A Family? A Constitutive-Affirmative Account

**DOI:** 10.1007/s11673-024-10339-x

**Published:** 2024-03-26

**Authors:** J. Y. Lee, R. Bentzon, E. Di Nucci

**Affiliations:** University of Copenhagen, Øster Farimagsgade 5, København K, Denmark

**Keywords:** Family, Parenthood, Assisted reproduction, Bioethics

## Abstract

Bio-heteronormative conceptions of the family have long reinforced a nuclear ideal of the family as a heterosexual marriage, with children who are the genetic progeny of that union. This ideal, however, has also long been resisted in light of recent social developments, exhibited through the increased incidence and acceptance of step-families, donor-conceived families, and so forth. Although to this end some might claim that the bio-heteronormative ideal is not *necessary* for a social unit to count as a family, a more systematic conceptualization of the family—the kind of family that *matters* morally—is relatively underexplored in the philosophical literature. This paper makes a start at developing and defending an account of the family that is normatively attractive and in line with the growing prevalence of non-conventional families and methods of family-formation. Our account, which we call a *constitutive-affirmative* model of the family, takes the family to be constituted by an ongoing process of relevant affective and affirmative relations between the putative family members.

## Introduction

Contemporary Western societies continue to value the nuclear family—composed of a heterosexual couple with genetically related offspring (Williams 2011, 1026)—as an ideal family structure. Implicit in this traditional nuclear model is an endorsement of both *bionormativity*—the valuing of biological^1^ relatedness between family members—and *heteronormativity*—the valuing of monogamous, heterosexual partnerships as a foundation for “the family” (Overall 2014, 2; Witt 2014). This so-called nuclear structure depicts a rather narrow and specific picture of what a family might look like. As Robert Wheaton points out, for instance, historically “no European Christian societies are known to have practiced polygamy, concubinage has been very rare, and there are no documented instances of matrilineal organization” (Wheaton 1975, 608).

It is clear, however, that the bio-heteronormativity of the nuclear structure is not *necessary* for persons to belong to the same family—at least, not for the kind of family that *matters* to people. Divorce rates, unmarried cohabitation, single parenthood, have all risen in our age (OECD 2011, 7), and it is increasingly commonplace for people to form families in diverse ways that deviate from the nuclear ideal. For example, one might acquire family members through re-marriage, utilize donor gametes to produce offspring, and opt for adoption rather than procreation, to mention a few alternatives. Scholars across the social sciences, in which the family is a basic unit of study (Sharma 2013, 306), disagree over the evolving definitional boundaries of family structures (Lubbe 2007, 262), and have long recognized that the traditional view is subject to critique. As Kathleen Gough hypotheses, any past of the family does not “limit its future … neither the family itself nor particular family forms are genetically determined” (Gough 1971, 770).

Yet families whose structure deviates from the bio-heteronormative ideal are still frequently stigmatized or discriminated. Alternative family structures can be, for example, *legally* discriminated against (Weisberg 1975, 555) by their being “denied benefits which society bestows upon families which resemble the traditional model” (Treuthart 1990, 92). That is, the law might prevent more than two parents from being recognized as parents (Lewis 2016, 745) or prevent same-sex partners from access to certain provisions available to the “traditional” couple, due to their being unmarried, for example (Duncan 2001, 59). We might also recognize routine examples of ways in which “families” are measured up and judged against an implicit bio-heteronormative ideal: childless partners are frequently asked when they plan to procreate, many same-sex or LGBTQIA+ aspiring parents struggle to adopt children, surrogacy arrangements are presumed more desirable if there is a genetic link between at least one of the intended parents, and so forth.^2^

Of course, if we restrict the meaning of the family to merely prudential terms, for instance as a type of economic relation, a way to benefit from shelter and a household, a source of cheap labour, and so on (Kleingeld and Anderson 2014, 322), then perhaps no thicker account of the family is needed. However, if we are at all interested in the notion of families based on “members taking themselves to be part of a special relationship … acting on the basis of affection, and sharing a concern with the long-term well-being of the family as a whole” (Kleingeld and Anderson 2014, 322), then it is indeed imperative to spell out what *more* the family is, beyond a shallow description of members who are said to count as a family unit. By identifying an account of family that captures these meaningful relationships, we might therein find the resources to distil the value of the family without designating only its traditional *forms* (like the nuclear family) as desirable.

Scholarly attempts to better capture what is normatively desirable about the family—without at the same time *limiting* who is included in the family picture—have already been made: legal scholars, for instance, have argued for a “functional” definition of the family, rather than a natural or traditional one, based on the instrumental value of the family as one that provides “love and support to its members” (Treuthart 1990, 92). This is meant to ensure that social units that *function* as a family are legally protected—those who are unmarried or otherwise fall outside of the traditional model could be included. Similarly, sociologists of the family have noted that the family serves “key functions” that contribute to the overall flourishing of a society, such as companionship, economic provision, shelter, and so on (Blackstone 2014, 53).

So what contemporary contributions have philosophers made on the topic of family? In moral and political philosophy, scholars have engaged in debates about the value of the family from a parenting and parent–child relationship perspective especially. Ferdinand Schoeman, for instance, has said that *intimacy* is an essential part of “the basis of the parents’ moral claim to raise their biological offspring in a context of privacy, autonomy, and responsibility” (Schoeman 1980, 6). The importance of intimacy is also emphasized by Laura Kane’s view: Kane proposes that “we conceive of family … as a social group that is based upon a commitment to interdependent caring relations and the fulfilment of mutual well-being through those relations” (Kane 2019, 77). In laying out this proposal, Kane highlights intimacy and care as *primary purposes* of the family. Finding the primary purpose of a social group is a useful methodological device to separate and identify different groups. Harry Brighouse and Adam Swift have pointed out that parents have a “power of life or death over their children” (Brighouse and Swift 2006, 92). In their book, *Family Values: The Ethics of Parent–Child Relationships* (Brighouse and Swift 2014), they discuss that it is generally a valuable thing for children to be raised by parents insofar as familial relationship “goods” are conferred. (Brighouse and Swift 2014). Anca Gheaus has defended rights to parent based on children’s interests (Gheaus 2021, 434) and the special moral status of the family as being based on embarking on a certain kind of commitment—including the commitment to raise children (Gheaus 2012, 122).

Yet, there remains a dearth of philosophical accounts of the family which serve to both *demarcate* family relationships from other kinds of relationships, and to also capture the *value* of families. Our article endeavours to make a contribution by proposing the foundations of an account which we call the *constitutive-affirmative account*, in which the family is, simply put, conceived of as an ongoing process of the relevant affective and affirmative relations. Our defence of the account proceeds as follows: Section "Different Types of Families" begins by providing examples of families that deviate from the bio-heteronormative ideal. In Section "The Constitutive-Affirmative Account: Beyond the Bio-Heteronormative Ideal", we spell out the features of our constitutive-affirmative account, which will serve to explain why the examples from Section "Different Types of Families" indeed constitute families. In Section "Objections", we address potential objections to our account, and defend our view against them.

## Different Types of Families

In the following we present several different examples of family forms which deviate from the bio-heteronormative “ideal.” The examples included cover a range of possibilities. Our hope is that these examples illustrate the broad but plausible scope of what families *can* be, before putting forward our constitutive-affirmative account and elaborating on how our account would be inclusive of the following examples.

### Multi-Household Families

Because interventions like reproductive technologies are now more widely available, it appears more commonplace for a greater number of aspiring parents to be involved in the family-making process, and for families to be composed of multi-household units. Take this excerpt from Claire, who is part of such a family:

I’ll give my best simplified description of our family: my mother, my half siblings’ mother and our father were friends living in the Bay Area in the ’90s. At the time, both women were in their 30s and wanted to have children—but neither had a long-term partner. My father, a gay man and also partnerless, agreed to be their donor and, if things worked out, involved in their children’s lives.

My brother was born in March 1997, followed by me in October of the same year, and my half sister came along three years later. As a child I got strange looks when I told people that my brother was seven months older than me. But I just thought of us as a family that happened to live in three separate households.

[Claire Huag, *New York Times*]

This testimony implies that “family” need not be limited to a single household, nor be exclusive to one partnership. Families might be created from a common cause, or joint purpose, which in this case involves child-rearing and being committed to support and be part of each other’s lives in a meaningful fashion.

### Platonic Co-parenting

Similarly, it is entirely plausible for new families to be created by people who are not necessarily romantically involved with each other but share in a common, meaningful project, such as raising children together. Platonic parenting, or “co-parenting,” is a term used to denote such an arrangement. Long-time friends who are childless but want to raise children without going through the steps of finding a romantic partner might opt for platonic co-parenting; but equally, this co-parenting arrangement can take place just between individuals whose primary common interest is to become parents.

In one example from 2014, a man named Charles Bourne set up a profile on *Modamily*, a site that enables people who want to have and raise children to connect. He was then contacted by a person on the site, Nisha Nayak, a forty-year-old psychologist. They proceeded to meet and get to know each other, after which Nayak underwent in-vitro fertilization in 2014 and conceived twins (Traverso and Robbins 2018).

Of course, platonic co-parenting does not just apply to *aspiring* parents who want to have and then raise children: while forming families by literally matching with potential co-parents appears novel or unusual, we do have more banal, commonplace cases. An ordinary case could consist of divorcees who share child-caring responsibilities after divorce. Negotiating co-parenting arrangements is not alien or novel, after all: after a divorce, practical details like custody and visitation rights are addressed. Although we might conceive of this latter example as an “end” of a family, the point is that re-negotiating boundaries need not imply that a group cannot still be a family in some meaningful sense, even if the heteronormative form has lapsed.

### Cohabiting Friends

A story from *The Guardian*, written by Andrea Hargreaves, describes how after seven years of living on her own, the author moved in with two other long-term friends, each of them selling their home and putting all their savings towards a new, big communal home. She says,

Of course we are resigned to being described as bonkers but we wear it with pride. Thanks to help from my two lovely friends, I hosted a party for 100 people to celebrate my 70th birthday only four days after we moved in, and a riotous 96th birthday party for my lively mother yesterday. We are all involved in a protest to restore our rail timetable, the kitchen is being remodelled, Sally-Mae’s studio is being converted and we are digging out raised beds for a kitchen garden. And that’s just today. A male friend of mine remarked, on seeing all this going on, that he didn’t think three men would be able to live as cooperatively as we three women. “They’d still be arguing over how to do it in a year’s time,” he said.

[Andrea Hargeaves, *The Guardian*]

While on a more traditional view of the family we might envision moving *out* from houses where we live with housemates or friends as the step into a more settled, family living, this story reminds us that having one’s own partner and children from a separate relationship does not exclude one from living in family-like arrangements with those who are not necessarily our conjugal partners or blood relations. These women indeed may have their own partners, as well as their own children, but they choose to live among friends. This is an example where it is clear that friendships can overlap with family relationships; friends can commit to living under one household, celebrate birthdays and other big days together, tackle and share in tasks both mundane and serious as a group, and in general undertake various communal projects as joint and shared endeavours.

## The Constitutive-Affirmative Account: Beyond the Bio-Heteronormative Ideal

An advocate of the bio-heteronormative ideal of the family might defend it by using identity-based reasons. David Velleman, for instance, has claimed that it is morally problematic to create children with anonymous genetic donors since it will harm the children not to know their genetic history. Velleman claims that biological family relations offer “ … a connection of the sort that normally informs a person’s sense of identity, which is composed of elements that must bear emotional meaning” (Velleman 2005, 363). Some may point out, however, that traditional perspectives on the bio-heteronormative family are already out of vogue given the increasing acceptance of the diverse methods we have for family-formation (and Velleman has indeed been already widely criticized within relevant literature, see for an overview: Di Nucci 2016). Yet there isn’t as much discussion, in the philosophical literature, of substantive alternatives to the bio-heteronormative ideal.

We endeavour to provide a philosophical analysis of the family which distils its value without at the same time inadvertently endorsing bio-heteronormativity. We call our view the *constitutive-affirmative account*, which we will state in the following way, and then work out the details in the subsequent paragraphs:

The kind of family that matters morally is constituted by a continuing process of the relevant *affective* and *affirmative* relations, conducted in a *reciprocal* and *ongoing* fashion.

Let us first discuss what is meant by the relevant affective and affirmative relations. Though our aim here is not to make an exhaustive list of necessary and sufficient conditions for the family, the items mentioned herein should give an idea of the kinds of relationships and social phenomena that might constitute a family.

An obvious candidate for *affective* relations involves the presence of pro-social affective leanings within the proposed family group, like emotions related to care and love. This can be interpreted latitudinally—love can manifest in diverse forms, some more or less obvious than others. The parent who signs up their child to multitudinous extracurricular activities might look like an overbearing parent to some, but this may be just as valid an expression of care over the child’s flourishing than a parent who says “I love you” frequently or another who is particularly permissive and open towards their children’s preferences (remember: we are here to offer an account of family and in this particular case parenthood, not of “good family” or “responsible parenthood”).

Although we have not exhausted the substantive content of the affective leanings that may constitute family relationships, our view is that affections of love, concern, and care are likely to be present or manifest in many forms, with the caveat that some of the relevant affective attitudes may not *always* be positively valenced (it may be that periods of indifference, for example, are part of the rich constellation of attitudes that could be taken toward family members).

As for the *affirmative* relations that might constitute a family, we have several relevant candidates. One aspect to consider is the role of deeply trusting relations as helping to mark out the proposed family group. When we think about who our families are, we might have in mind our emergency contact, or the person we rely on to help us out of a difficult situation when we are most vulnerable. The people we enlist and designate as those we trust enough to support us in situations or decisions of life-or-death magnitude, arguably reveal the “deeply trusted.” Trusting relations are not necessarily conducted in a completely equal fashion, however; trusting relationships may also involve dependant relations. By dependant relations, we refer to nurturing or care-taking relationships between parties who are differentially endowed in terms of what they might provide for the other parties. Just because a toddler does not speak, for instance, does not mean they do not affirm their reliance on another—when they cry out for a parent, they partake in affirmative relations where certain happenings or agreements are merely implicit but exercised just as effectively as relations where people have explicitly agreed to something.

Another aspect of affirmation is collaborative interaction and influence that involve identity-making and identity-shaping. When we set up lifelong commitments to our partners, or agree to certain conditions and obligations that we vow to help one another carry out, or decide to embark on transformative journeys with others (moving country, adopting together, buying a house, etc.) we collaborate with others in non-trivial ways. Such collaborations have the effect of shaping the very persons we are and become. Of course, we might conduct such interactions in spaces we consider more professional than family-like: when we undertake group assignments as part of our professional work, for instance. Typically, however, such interactions are not considered as part of a bigger whole—they are projects that stay in the job and do not necessarily bleed into one’s more private life and identity (unless, perhaps, we are talking about a family business).

In the family case however, we would say, the collaborative interaction conduces to a shared narrative *history*,^3^ a narrative-building, between the proposed family members, in which the obligations and duties generated within the shared narrative are particular, rather than general, and salient in ways that ad-hoc projects are not. For instance, leaving a job, rejecting a work assignment, and so forth, may in some cases evoke disapproval or be seen as unprofessional, but beyond the general moral banalities that oversee everyday interaction—etiquette, displays or respect, and so forth—there are no *particular* obligations that bind one to such commitments. This is not the case when it comes to family—decisions to pull out of a family-designated commitment, not help out, and so forth, have significant moral implications for the given relationship. The ongoing nature of collaborative identity-making between different parties mean that particular members come to share a unique narrative history and commit to build on that narrative together.

The final element we want to point out is the way we identify *as* and *with* family members. This means *identification* is just as important as the affecting of one another’s identity. When “I” consider myself my mother’s daughter, I am not merely referring to some descriptive causal relation, like being the female progeny birthed by a body genetically related to me. I am, on the one hand, talking about the way she shaped who I am (the identity-making mentioned prior); the way I tend to fill my spare time with creative endeavours, as she does, the irrevocable passions and tendencies that make up the person I am, and so on. These are all features of me that I was never asked to exhibit—yet they are me, because I am my mother’s daughter. Importantly, however, I am also voluntarily *identifying* as my mother’s daughter: I am *endorsing* the being I am, the result of me, as arising out of the relationship I have with my mother. By identifying as such, I capture a multifaceted attitude of endorsement—that I want to be *called* her daughter, and that I approve of that designation, for all the possible flaws and triumphs of my upbringing. This can be contrasted both with someone who is unaware of replicating their parents’ attitudes—say politically; or alternatively with someone who explicitly makes a point of abandoning some trait or conviction precious to their family—religion, for example. When we identify as certain family members, we place ourselves in a position whereby we endorse or take on roles that will be appropriate for those relationships^4^ (e.g., mother–daughter, siblings, etc.). These affective and affirmative ties are significant enough to inform some of an individual’s biggest life decisions, like which university a member would like to apply to, how far away they should move for a job, and so on.

Let us now clarify what we mean when we say that these affective and affirmative relations are to be conducted in a *reciprocal* and *ongoing* fashion. Implicit in the affective and affirmative conditions laid out above is the fact that such phenomena are legitimated and conducted within multi-directional relationships. Other scholars have also stressed the importance of reciprocity as a defining feature of families, e.g., Kane (2019, 2021) and Amy Mullin (2006). In relationships with reciprocal value, we often find ourselves conducting interactions and obligations that may not feel entirely *voluntary* to us, or something we can help, although they are neither repudiated.

For example, the trusting relations between carers and dependants are reciprocal because they invoke expectations of certain conduct from both sides. However, there is normally a skewed relationship between what the dependants need to flourish compared to their carers’ need. This unequal reciprocity need not be a problem for the constitutive-affirmative account, as Kane points to useful distinction: reciprocity can be “equivalent in intention” without being “equivalent in performance” (Kane 2021, 319). By this, we understand that what matters is what family members intend to, in order to provide care, more than what certain family members are capable of. This has the advantage of not excluding any members of the family with no capability of matching the level of care that they receive, e.g., infants that are not capable of caring for their parents. However, Amy Mullin points to the fact that infants do reciprocate the care from their mothers, just not in an easily recognizable way. A study by Megan Kirschbaum and Rhoda Olkin (2002) showed how babies with disabled mothers accommodate the mothers’ needs, for example by stiffen their body before being lifted, to make it easier to lift the baby with one hand (Mullin 2006, 184).

As for the *ongoing* nature of affective and affirmative relations, we refer to their persistent and all-encompassing nature—they are rarely thought of as temporary circumstances but rather they are long-term relations that carry significance for any agent member in all realms of their lives. This is why we may find it difficult to relate to cases where partners have already agreed that their relationship is temporary early on.

### Diverse Family Structures and the Constitutive-Affirmative Account

We are now in a position to explain how the various family forms in Section "Different Types of Families" constitute entirely plausible candidates for families, given the nature of the close relationships implied in them. The reason we have made a point of including less conventional examples of family structures in our article is to indicate that the *composition* of family members is not really what matters: what emerges above is that the nature of the *relationships* these different agents have with one another is crucial to what make them count as “family.” This is, in fact, already implicit in the more conventional cases of family ties: for instance, a nuclear family that goes through a divorce is not typically thought to automatically be discounted from being “family,” given that co-parenting provisions might be made thereafter, and an ongoing and significant relationship be maintained between the members. What we have endeavoured to do, then, is to reveal that there is no inconsistency or challenge involved in thinking of “unconventional” families as families—besides our prejudices or lack of familiarity with different family structures—precisely because families are constituted by the relevant affirmative ties themselves, rather than by pre-determined forms. It would be telling, for instance, if one were to respond adversely to any of the above examples but unquestioningly include distantly genetically related people one has never met as “family.”

Perhaps some may still be inclined to insist that the family is “merely” about the kinds of groupings that are used for collecting demographic data (e.g., information about household income, etc.) and that we ought not to quibble over the deeper meanings of the term. Yet, it does not seem that drawing the boundaries on the family this way really captures the colloquial and everyday sense of the concept, which seems to involve a thicker meaning of the kind described in our constitutive-affirmative account. For example, sayings like “They are like family to me,” “I have duties to help my family,” and so forth, capture the fact that family relationships are the kinds of relationships that have moral value on an individual and interpersonal level. Counting household members or merely looking at marriage certificates, then, might tell us a little bit about how family members conduct themselves (they might get married, for instance). But these descriptions are not *ultimately* the kind of thing that can measure the full *extent* of the ways that deeply involved relationships can be identified as familial and hold great meaning for people. It seems only that something like a constitutive-affirmative account, which by default emphasizes the nature and quality of the relationships conducted, rather than the *form* of the group unit or the composition of the candidate members, is equipped for the task of *substantively* determining what the family is.

## Objections

In this section, we anticipate several challenges to the constitutive-affirmative account. By anticipating and addressing these potential objections, we will hopefully strengthen and enrich our account.

### Elective Family

One might wonder whether our constitutive-affirmative account makes the family an *entirely* “elective” or voluntary endeavour and also whether that would make it a *problem* for our account. Let us consider three major issues that might be levelled at an *entirely* elective family account, and clarify why this would not be such a big issue even if our account were to be taken to present a voluntarist view of the family:

1. The first issue is that if the family is a matter of purely voluntary association, it might enable members to shirk duties that seem plausible to ground as morally obligatory on the basis of family membership. For example, we might be uncomfortable with the idea of, say, a father who does not wish to pay child support by trying to argue his way out of “family” status: he might claim that he never wanted the pregnancy to go forward in the first place, that he is not in a significant relationship with the other partner nor attached to the child, and so forth. In short, one might argue that the affective and affirmative dimensions of a family relationship do not hold for their case, which they then use to justify why they ought to be released of family-related duties as a consequence. What should we make of a claim like this? Firstly, we could respond here that a call for greater state support of parents with dependants is needed, rather than to insist that this issue calls for a return to the more “traditional” forms of family where familial duties are designated according to certain norms (e.g., that a male “head” of a family unit must fulfil their role as provider of the entire family). Additionally, however, we would push back on the thought that a voluntarily associating family group is simply *free* to abandon the commitments they have made within that arrangement. Many would agree that people who rely on each other for support, such as long-term friends or partners, have moral obligations to one another, even if there is no *right* or *law* that officially binds these agents’ obligations to one another. Reasonable expectations that have been established in view of certain relationships seem plausible to upkeep and maintain even in the absence of some officiated bond. Regardless, it’s not as though those who *are* legally or by blood bound together are immune to suffer abandonment or neglect from family members.
2. Secondly, we might worry that elective or voluntary family membership negatively implicates those who are most vulnerable to suffer exclusion. One thing that we might find positive about *involuntary* accounts of family—that designate family members not because of the nature of the relationships involved but rather according to some pre-determined relation (e.g., blood ties)—might be that those who are most difficult to love and take care of are still *included* in a group that shares in some benefit, e.g., a common shelter. So a difficult relative could not simply be shunned on the basis that everybody else finds them annoying, a parent could not simply abandon their child if the latter’s disabilities became too burdensome to handle, and so forth. It is not obvious, anyway, that a more (supposedly) voluntary account of family should permit people to cherry pick who to include and exclude. When one is a primary caretaker or dependant, for example, we would plausibly say one is duty-bound to stick around. It is not simply because one is a biologically-related mother or father that one is duty-bound not to abandon their child; it is because one might be solely entrusted to know and attend to the child’s needs and interests, because one cares about their child’s welfare, because one takes oneself to be responsible over the child, and to care about the child, that one would feel compelled to endure the more difficult and challenging dimensions of the bond. Electively associating as a family member would not thereby make it more permissible, or even easy, for people to drop family members at will, given that the way our narratives intertwine with those we know deeply, have grown up with, have provided for, and so forth, are not necessarily entirely voluntary aspects of the family relationship but rather the *irrevocable* aspects of the affective and affirmative relations that constitute them.
3. There is, however, a third worry that might arise in relation to the more elective nature of the constitutive-affirmative account. The worry is that a constitutive-affirmative account is overtly positive, and requires only *good* relations to hold between family members. If this is the case, there would be no such thing as *dysfunctional* families—there could only be families and non-families. To this, we would point out several replies: first, the constitutive-affirmative account is only positive in the sense that certain cares and interpersonal narratives must be *present* in a family relationship. One may have serious arguments and profound disagreements with family members, and still be *invested* and *caring* in the appropriate ways to constitute a family; indeed, the conflicts that sometimes surface in family relationships may even be characteristic of how involved and communicative the family wish to be with one another. These hardships may together shape the lives and identities of the family members who manage or overcome these challenges—and this growth, too, can be a valid aspect of constitutive-affirmative relationships. A similar argument can be found in Kane (2019), where she argues for a normative definition of family, thereby making it impossible to be in a “bad” family (Kane 2019). Our perspective on this, however, is that if the family group is *so* dysfunctional as to seriously harm its members, or if the family group become *so* estranged and distant that its members no longer know each other very well, and so on, there is no reason in principle to oppose their no longer counting as a “family”; at such points, it may be even painful, and not very accurate, to designate certain groups as families anyhow.

### The Problem of Ontological Messiness

Following on from the potential problems for an elective family above, one might say that the constitutive-affirmative account is ontologically messy. One might claim, for instance, that this view of the family lacks identity over time, given that the family must be actively constituted as a process of affective and affirmative ties, which are not fixed. This might seem counter-intuitive for those who believe that the distinctiveness of family consists in its hardiness: families endure because they consist of entirely involuntary ties that are nigh impossible to be rid of or dissolve (for instance, it is impossible to “undo” being the birth mother of some child, or to get rid of a genetic tie we may hold with some relative).

In addition, one might also argue that this account brings too many families *into* existence. For instance, sorority and fraternity groups might come to be real families; on the other hand, too many families might have to go out of existence, like distanced blood relatives. In our view, this is not so much an objection to the account as it is unfamiliar territory. When families split up, divide their assets, or become estranged and go on to identify with “new” families—all very common enough occurrences in our world as is—the question of identity over time, as well as which units are to count as “family,” also become significant. Yet if we can acknowledge it is possible to create new families—as when two once-perfect strangers get together and become married, when we acquire new siblings, and so forth—we should wonder why concerns over the stability of the family identity only surface in cases where the family-formation is unconventional.

Furthermore, although we would acknowledge that the account might carry the effect of seemingly conjuring new groupings of family in and out of existence, we do not view this as a morally troubling issue; in fact, here too conceptual inclusiveness is only a good thing. Perhaps there are indeed “family-like” relationships, which ought to be recognized as such, like when a group of people share a significant set of life experiences together. On our view, there are grounds to recognize such groups as families. On the flip side, our account can accommodate the view that extremely dysfunctional families are not really like families—at least not in the morally valued sense of the term. For families that contain neglect or abuse, there does not seem to be good moral reason to *stay* a family.

### The Problem of Overlap With Other Social Relationships

Another worry may be that the constitutive-affirmative account overlaps too much with other kinds of social relationships, making it unclear how we ought to distinguish our account of family relationships from other ones. Our response to this—as hinted at in the section above addressing the ontological messiness of the family—would be to say that the family is not a finalized condition but rather contiguous with other valuable social relations.

The demarcation process for the family need not be strict—we can allow for there to be a kind of gradient where people can be *more* or *less* family-like by degrees, as well as allow former friends to become family members and former family members to become non-family members. Yet, we do not believe this to make the account insufficiently explanatory when it comes to drawing boundaries between different kinds of social relationships. The constitutive-affirmative conditions outlined capture what is *typical* of family relationships, rather than other kinds of relationships, but this does not bear on whether other kinds of relationships can take on family-*like* characteristics (see also Kane 2019, 2021). We believe the conditions for a constitutive-affirmative approach lay out a plausible enough picture of how this might be possible—for instance, much like family relations, long-standing and profound friendships may well be treated as a primary site of identity-shaping, narrative history, trust, and so forth. This is not the case between newer friends or mere acquaintances. As such, our view is perfectly in line with the intuition that the development of deeply involved relations of a family-like level takes quite a bit of effort and work.

### The Problem of Practical Reform

What does it matter that a constitutive-affirmative account lay out the *moral* conditions for the family if in reality the legal sanctity of the family remains firmly rooted in the nuclear bio-heteronormative ideal? It may be all well and good to endorse a constitutive-affirmative account to cover the everyday sense of the family concept, to describe the relationships people find valuable, but we might doubt whether this sort of account has any practical usage. Our view is that the constitutive-affirmative account ought to inform the legal and policy landscape as well. Recent advances on policies related to validating diverse family fronts have been made in, for instance, allowances for lesbian couples to seek state funded IVF treatment or for more than two parents to be declared legal guardians. The constitutive-affirmative account is entirely aligned with such changes.

Another question that might come up in terms of practical reforms to family recognition and protection regards whether it is not practically more desirable just to eliminate the category of “family” altogether and to try and find better designations to protect and benefit people, given that the law may only protect or privilege families that conform to a certain *form*. We might wonder whether such benefits—say, tax breaks only for married couples—should not just be gotten rid of all together, so that *all* members of meaningful family-like relationships can benefit in similar ways without having to commit to a specific constellation of family members. In the context of marriage, for example, Clare Chambers has argued for the ideal of a marriage-free state in which the term “marriage” would, like friendship, denote a certain kind of relationship, but not be “a matter of legal ruling” (Chambers 2017, 3). Of course, there are existing policies we might work on changing slowly, as we are not starting from scratch, and perhaps we might hope one day that “families” are only identified according to the nature of their relationships rather than by some official designation preferred by the state or by prudential relations. But this objective, we think, would make it more, not less, imperative to lay out a proper conceptual foundation of the family that philosophically considers the value of the concept.

## Conclusion

We hope to have motivated the need for further conceptualizations of the family that go beyond the bio-heteronormative default. We proposed the *constitutive-affirmative* account as a starting point for an alternative philosophical view that emphasizes the relevant affective and affirmative relations as constituting the family. In so doing, we endeavoured to articulate an account that is plausible and normatively attractive (relative to bio-heteronormativity) as a theory of the family and its value. We also dealt with potential counterarguments that might be levelled at our account and responded to a range of objections. On the whole, we hope that our account can contribute explanatory value as a framework equipped to challenge and disrupt prevailing norms about family ties.

## Footnotes

1 By biology we mean to cover all the possible ways to be biologically related to someone. Therefore, this covers both genetic donors and parents as well as gestational parents (see, for example, Di Nucci 2016).

2 There is a recent example on this, from Denmark: two men cannot both be formally recognized as fathers.

3 While others may argue that genetic relatedness or something of the sort is what connects family members in this sense, we would argue that the narrative history does not necessitate ties beyond the relationship itself. Here, we see that our theory can provide an answer to some of the considerations from the bio-heteronormative theorists: identity-forming is an important part of a flourishing life, and this do and should happen in the family setting. However, the identity-forming might not have any necessary relations to the biological component of some families – as mentioned above, we see many routes to forming an identity.

4 See also Laura Wildemann Kane on collective intentionality (Kane 2021).
